# A rare case of an unexpected trigger of paroxysmal atrial fibrillation in the right atrial appendage diverticulum

**DOI:** 10.1186/s12872-024-03789-z

**Published:** 2024-02-29

**Authors:** Li Cheng, Jianbo Hu, Yuanping Zhang, Zhaohua Geng, Bo Zhang

**Affiliations:** 1 Department of Cardiology, Songshan General Hospital, 69 Xingguang Street, Chongqing, 401120 China; 2 Department of Cardiology, Second Affiliated Hospital (Xinqiao Hospital), Army Medical University, Chongqing, China

**Keywords:** Right atrial appendage diverticulum, Paroxysmal atrial fibrillation, Catheter ablation, Surgical intervention

## Abstract

**Introduction:**

We described a rare case of an adolescent girl with paroxysmal atrial fibrillation originating from the right atrial appendage diverticulum and successfully converted to sinus rhythm after surgical intervention.

**Methods:**

A 19-year-old girl was referred to the hospital for a catheter ablation of paroxysmal atrial fibrillation. conventional radiofrequency ablation using 3-D mapping were ineffective. Activation mapping showed the root of the free wall atrial appendage was first excited and catheter modeling (3D Carto map) showed a sac-like structure.

**Results:**

We did selective angiography and further Computed tomography angiography (CTA) and Transesophageal echocardiography (TEE) which showed diverticulum originating from the right atrial appendage. Hence the patient was referred to cardiac surgery and had no recurrent atrial fibrillation at three months postoperative follow up.

**Conclusions:**

Right atrial appendage diverticulum was an extremely rare malformation that can coexist with atrial tachyarrhythmia. Surgical ligation or excision of the abnormal structure with local ablation can achieve excellent results.

## Introduction

Cardiomyocytes in the left or right atrial appendage may have abnormal increases in excitability and increased autoregulation for various reasons, leading to the development of atrial tachycardia [1]. Atrial tachycardias originating from atrial appendages most occur in young people and tend to present as persistent episodes with marked diurnal variations in heart rate, predisposing them to tachycardia-mediated cardiomyopathy [2].

## Case report

**Fig. 1:**
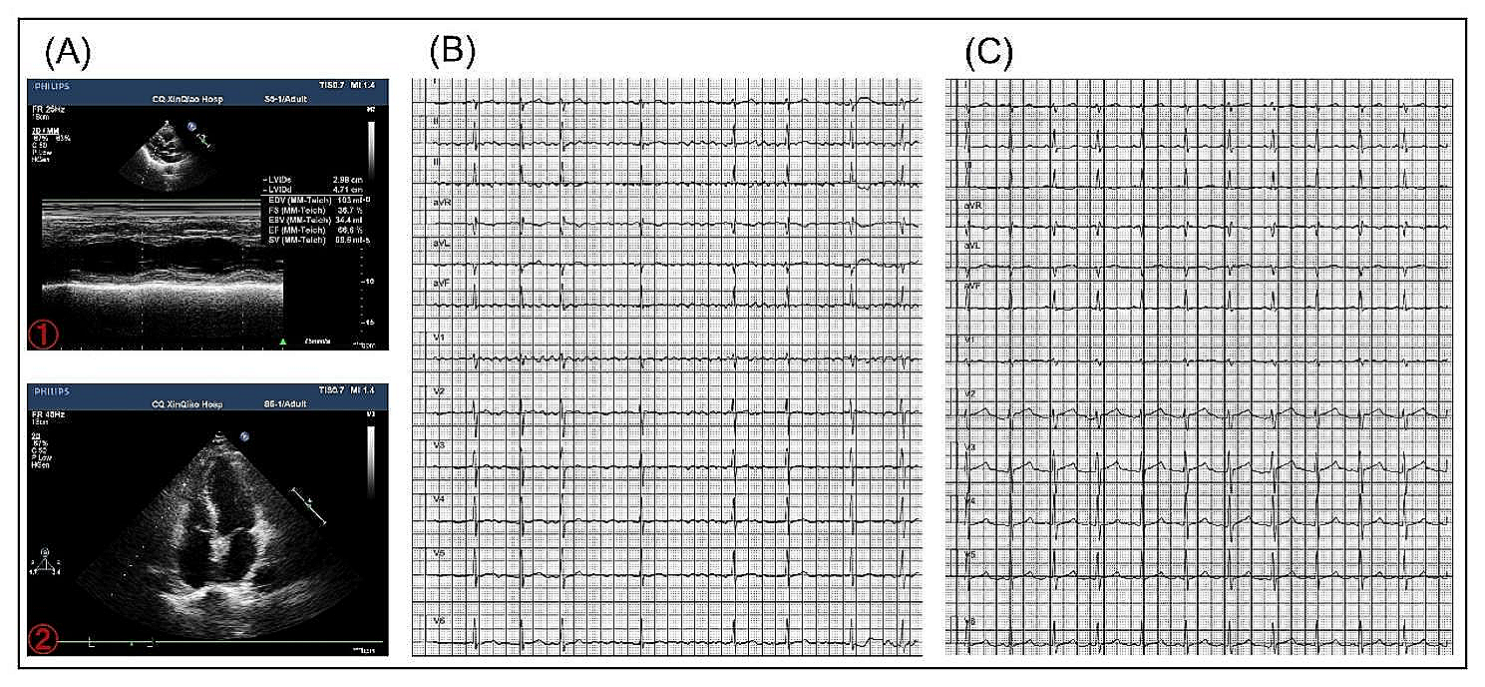
(**A**) Echocardiography showing normal diameter and function in all 4 chambers. (**B**) Twelve-lead ECG showing atrial fibrillation before surgery. (**C**) Twelve-lead ECG showing that the patient returned to sinus rhythm after surgery. ECG: electrocardiogram

A 19-year-old girl encountered the symptom of palpitation for 3 months with increased frequency and decrease in exercise duration. The ECG (electrocardiogram) showed atrial fibrillation (Fig. 1B) and several antiarrhythmic drugs were ineffective (including metoprolol, propafenone and amiodarone). Echocardiography showed normal diameter and function in all 4 chambers with mild tricuspid regurgitation (Fig. 1A), and transesophageal echocardiography excluded thrombus in the left atrial appendage. Regular examination ruled out hyperthyroidism and other diseases.

**Fig. 2:**
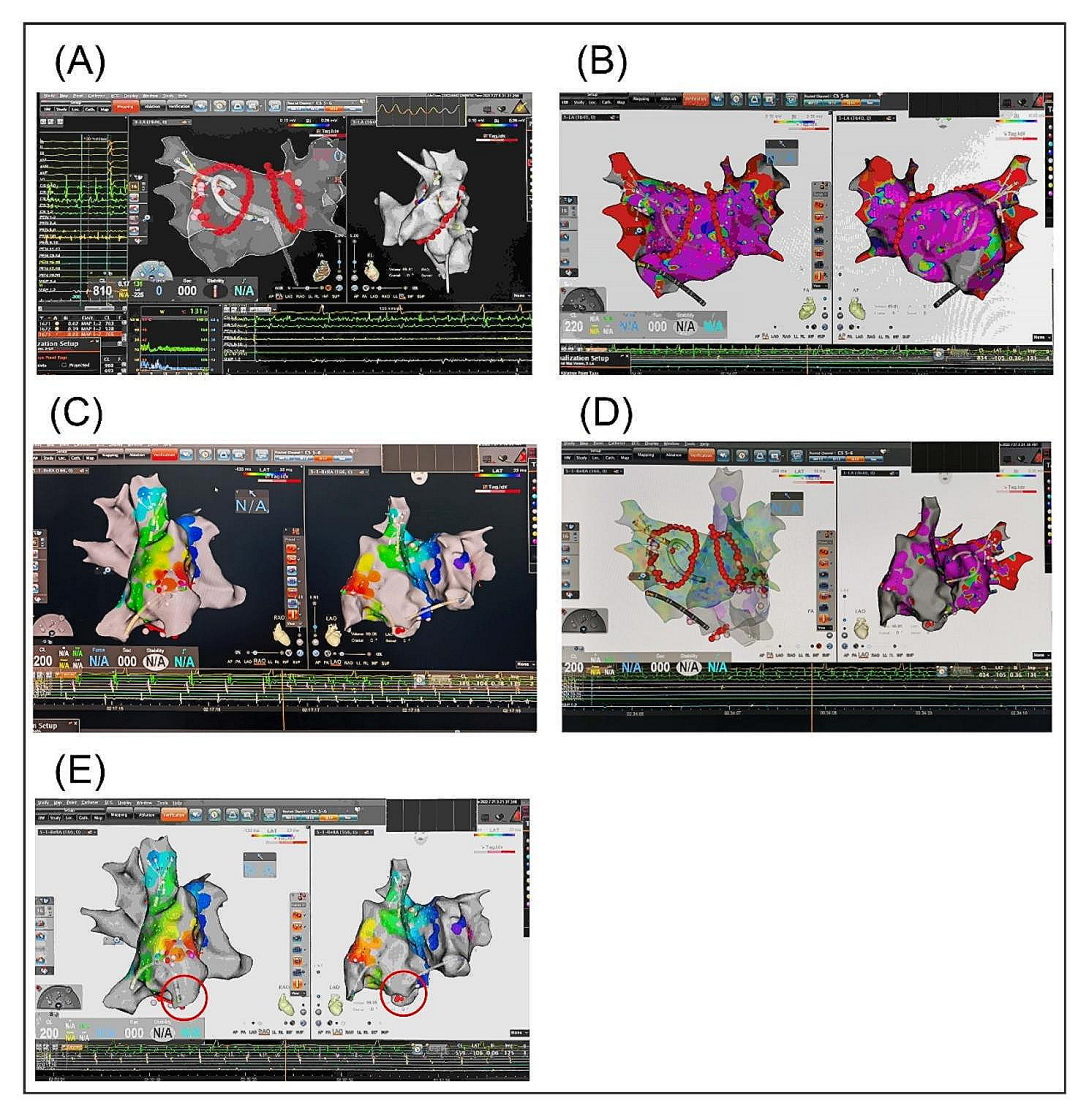
(**A**) Circumferential pulmonary venous isolation and no potential in the pulmonary vein mapping. (**B**) No obvious low voltage area and scar were found in atrial matrix mapping. (**C**) The root of the free wall atrial appendage was first excited in activation mapping. (**D**) Local site ablation failed to terminate the atrial tachycardia. (**E**) Catheter modeling shows a sac-like structure (the red circle)

**Fig. 3:**
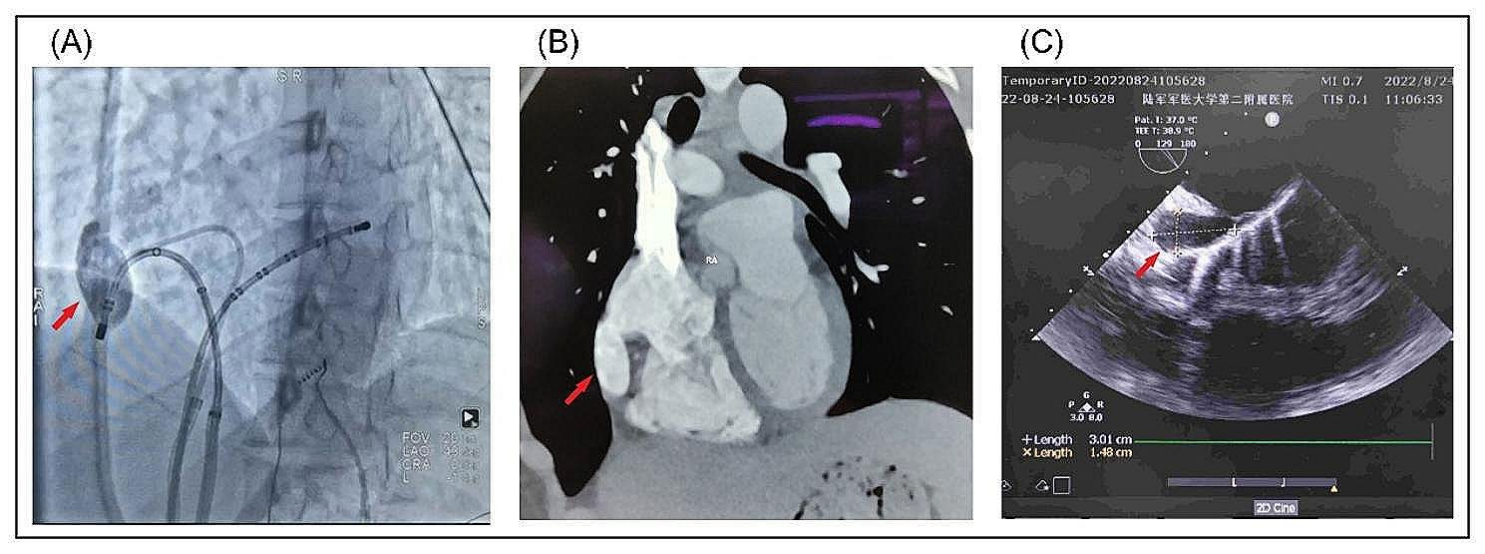
(**A**) Selective angiography showing a smooth sac-like abnormal structure in the right atrial free wall with no commissural muscle showing (red arrow). (**B&C**) CTA and TEE showing a diverticulum originating from the right atrial appendage (red arrow). CTA: computed tomography angiography; TEE: Transesophageal echocardiography

After Circumferential pulmonary venous isolation, there was no potential in the pulmonary vein mapping, but the intracavitary electrogram still showed atrial fibrillation (Fig. 2A). No obvious low voltage area and scar were found in atrial matrix mapping (Fig. 2B). Two hundred joules synchronized electrical cardioversion was performed, we observed 90ms advancement of the CS recording with initiation of the tachycardia, considered right atrial tachycardia, and activation mapping showed that the root of the free wall atrial appendage was first excited, where the catheter induced atrial fibrillation at this location, considered atrial tachycardia and atrial fibrillation originating from the root of the free wall of the right atrial appendage (Fig. 2C). Local site ablation failed to terminate the atrial tachycardia, the activation mapping was the same as before (Fig. 2D), then the right atrial fine mapping was performed and atrial fibrillation was triggered several times during the free wall mapping. Catheter modeling shows a sac-like structure, considering a possible right atrial free wall diverticulum (Fig. 2E). Therefore, a right atrial angiogram was performed, which showed a smooth sac-like abnormal structure in the right atrial free wall, with no commissural muscle showing, and the origin of atrial fibrillation was considered to be there (Fig. 3A). Was it right atrial diverticulum or variant right atrial appendage? Hence, we performed a CTA and TEE (Transesophageal echocardiography) which showed a diverticulum originating from the right atrial appendage (Fig. 3B&C).

Based on the previous findings the patient was referred to cardiac surgery and atrial appendage diverticulum was confirmed. After ligating the base of the right atrial appendage diverticulum and ablated the lateral fibrous ring at the base of the diverticulum and the right atrial appendage, the patient’s rhythm returned to sinus rhythm after 150 J synchronized electrical cardioversion, During the three-month follow-up, the patient had no recurrence of atrial fibrillation (Fig. 1C).

## Discussion

Diverticulum occurring in the heart are very uncommon cardiac malformations, and right atrial diverticulum are even rarer. In the current, the embryological origin of these diverticulum remains unclear, but they may be associated with other congenital cardiac malformations, and the prevalence is unknown because these rare abnormalities may be underreported, as some patients may be asymptomatic [3]. In a review of 103 patients with congenital right atrial and coronary sinus malformations, 17 patients presented with single or multiple right atrial diverticulum and 71% had clinical symptoms, which were mostly caused by arrhythmias [4]. In addition, the WPW syndrome has been described in some reports due to the entry of the ventricular myocardial bundle into the diverticulum cavity of the right atrial. These abnormal structures are clearly potential substrates for the origin of the arrhythmia [5].

Early recognition of right atrial appendage diverticulum is important but difficult, because of the rarity and concealment of this congenital structural malformation. However, the p-wave morphology of such patients is characteristic, mainly negative in lead V1 in all patients, becoming progressively positive across the precordial leads, and low amplitude positive in the inferior leads in the majority of patients [1, 5, 6]. This characteristic presentation can serve as a pointer to the patient’s examination and alert the imaging physician to these often unnoticed congenital structural malformations.

In the present case, we report a patient with atrial fibrillation of right atrial appendage diverticulum origin, but we did not perform further ablation because the safety and efficacy of ablation of the diverticulum itself is unclear. In some studies, mere point ablation of the trigger lesion of the atrial appendage is ineffective, whereas a strategy of electrical isolation of the atrial appendage plus progressive atrial ablation has been shown to have a long-term success rate of more than 80% [5]. Hikmet et al. have presented a case of successful ablation of RAA tachycardia using a cryoballoon. However, because cryo-catheter ablation requires close contact with the tissue surface and a very stable catheter position, there is a definite requirement for the morphology of the patient’s atrial appendage in order to create a large lesion [7]. But, from the other side, if atrial appendage was isolated, there was an increased risk of thrombosis of the atrial appendage and diverticulum, and patients are at risk of long-term anticoagulation with medication. However, it is clear that surgical ligation or excision of the abnormal structure with local ablation can achieve excellent results, as the right atrial diverticulum with complex connections is a rare but latent focal source of atrial ectopic beats triggering atrial fibrillation [8].

## Conclusion

Right atrial appendage diverticulum is an extremely rare malformation that can coexist with atrial tachyarrhythmias and lead to tachycardia-mediated cardiomyopathy. The safety and efficacy of ablation of the diverticulum itself is unclear. This report suggests that surgical ligation or excision of the abnormal structure and local ablation can be highly effective.

## Data Availability

The datasets used and/or analyzed during the current study are available from the corresponding author upon reasonable request.
